# Barriers to husbands’ involvement in maternal health care in Sidama zone, Southern Ethiopia: a qualitative study

**DOI:** 10.1186/s12884-019-2697-5

**Published:** 2020-01-06

**Authors:** Wondwosen Teklesilasie, Wakgari Deressa

**Affiliations:** 1 School of Public and Environmental Health, College of Medicine and Health Sciences, Hawassa University, Hawassa, Ethiopia; 2 Department of Reproductive Health and Health Service Management, School of Public Health, College of Health Sciences, Addis Ababa University, Addis Ababa, Ethiopia; 3 Department of Preventive Medicine, School of Public Health, College of Health Sciences, Addis Ababa University, Addis Ababa, Ethiopia

**Keywords:** Barriers to husband involvement, Maternal health care, Sidama, Ethiopia

## Abstract

**Background:**

Husbands’ involvement in maternal care is considered as a crucial step in scaling up women’s utilization of the services. However, the factors related with how husband’s involvement in maternal health care have hardly been studied to date in the study areas. Therefore, this study aimed to explore barriers to husbands’ involvement in maternal health care, in Sidama zone, Southern Ethiopia.

**Methods:**

The study employed a qualitative method. A pre-tested interview guide questions that prepared in English and translated in to Amharic language were used for data collection. The data were collected using focus group discussions, in-depth interviews and key-informants’ interview in April and May 2015. The data were analyzed thematically.

**Results:**

The study identified a range of factors that-deterred husbands to involve in their female partners’ maternal health care. These are childbirth is a natural process, pregnancy and childbirth are women’s business, preference for TBAs’ care and husband’s involvement in pregnancy and birth care is a new idea were identified as barriers for husbands’ involvement in maternal health care, in this study.

**Conclusions:**

A range of factors related with clients’ and service delivery factors’ were identified as barriers to husbands’ involvement in maternal health care. Based on the study findings we recommend a contextual based awareness creation programs about husbands’ involvement in maternal health care need to be established.

## Background

Husband (male partner) involvement is an important strategy for improving maternal health [1]. Positive husband involvement refers to the mental and physical participation of husbands in maternal and prenatal health and family planning in such a way as to increase maternal and infant survival rates and improve family planning outcomes [2]. Husbands’ involvement in maternity care, especially, starting from conception, throughout pregnancy, childbirth and PNC periods has a positive influences on birth outcomes [3, 4].

However, globally, men’s role in reproductive and sexual health has not been so widely recognized [5]. Particularly in most developing countries with low utilization of maternal health care (MHC), the concept of husband involvement is not generally accepted by people, as it is evident in literature [4, 6–10]. In developed nations, even with high utilization of maternal health care, husbands’ involvement is almost a norm, and its benefits are well articulated [3, 4].

The maternal mortality ratio in Ethiopia is among the highest in the world, which is 676/100,000 live births [11]. One reason is low utilization of skilled birth attendants during birth. About 90% of childbirths occur at home without skilled birth attendants assistance [11]. Among the reasons given for not using health facility during birth were ‘health facility was not necessary to give birth’, ‘it was not customary’ and ‘health facility was either too far or that they did not have transportation’ [11]. Moreover, as husbands are the heads of households, most of the decisions are mainly made by men and they control household expenditure in Ethiopian community. Thus, men may have crucial role in the access of mothers’ to timely and skilled care. According to EDHS 2016 report, high proportion of women reported that their husbands were one of the major factors for non-attendance of ANC during pregnancy [12].

In Ethiopia, a number of policies and strategies have been designed to improve the current situation of utilization of skilled attendants at birth, of which increased accessibility and availability of services at the community level is one [13]. Moreover, increasing deliveries attended by skilled attendants from 18 to 60% is a target that has been set in the Health Sector Development Plan IV (HSDP-IV for 2015–2020) [14, 15]. Those policies and strategies, however, have not given an emphasis to husbands’ involvement in maternal health care, even though it is a crucial step in scaling up women’s use of skilled prenatal care and then, improving maternal and child health [7]. This is supported by previous studies in Ethiopia and Nigeria that demonstrated the essential roles of husbands in prenatal care to promote the health of pregnant mothers and infants, and reduce maternal and infant mortality during pregnancy and delivery [16, 17].

In Ethiopia, in general, research evidence about factors that hinder husband’s involvement in MHC is limited. Therefore, this study tried to explore the main barriers to husbands’ involvement in maternal health care in Sidama zone, Southern Ethiopia. The findings will help to formulate policy guidelines and to design contextual based husbands’ involvement programs in maternal health care aiming to improve women’s utilization of maternal health care.

## Methods

### Study design

We used a qualitative study to explore ideas and experiences from men and women in the study areas. Focused Group Discussion (FGD), In-depth interview (IDI), and Key informants interview (KII) were used for data collection technique. The number of participants involved per FGD was between eight to twelve [18, 19]. Regarding the number of FGDs, IDIs and KIIs were determined based on the generated information from time to time until it becomes saturated [20, 21].

### Study setting

Sidama zone is one of the 14 zones and 13 special weredas of Southern Nations Nationalities Peoples Region (SNNPR), which were divided in to 21 woredas [22, 23]. The study was conducted in a purposely-selected five weredas of Sidama zone. The total number of population in the zone was estimated to be 2.9 million; of which, 6.7% are urban inhabitants [24, 25]. Women of the reproductive age group (15–49 years) and children under 1 year of age were estimated to be 23 and 3% of the total population, respectively [24]. In 2015, there were 7 primary hospitals, 1 general hospital, 127 health centers and 524 health posts in the zone. According to Mini-EDHS 2014 report, ANC, delivery care and PNC (within 2 days after birth) coverage of the region in the 5 years preceding the survey were 39, 11.7 and 11.1%, respectively [25].

### Study participants

The study participants for FGDs and IDIs were purposively selected samples of men and women from usual residents in the studied kebeles who fulfilled the inclusion criteria.

### Inclusion criteria for focus group discussions and in-depth interviews

- A man or a woman in a household, who were residents of the study sites for a minimum of 12 months, age > 18 year-old for men and females, married, a man whose female partner or a women had had a history of pregnancy or had at least one child in the past 5 years preceding this study were included in FGDs.
- Individuals who were found in the health facilities (men or women, but not from the same couple) during the data collection periods were included in the IDIs.
- Head of government or non-government institutions were included for KIIs.
- The study participants for KIIs were also purposively selected samples of stakeholders from zonal and wereda health offices such as maternal and child health program officers, chairpersons of gender’s offices, community leaders, religious leaders, traditional birth attendants, health extension workers, chairperson or secretary of women’s and youth’s Bureaus, head of health centers and midwives from maternity units were included. Health care providers who had had 1 year of or more work experiences in the maternity care department were interviewed individually.

### Recruitment procedures of study participants

We used a purposive sampling technique to select participants for the study. We used homogeneous sampling for FGDs, which mean, sampling people with a common identity to discuss their shared experiences. First, we selected five weredas in the zone. Secondly, two accessible kebeles per wereda were selected. Then, potentially eligible participants were identified and listed using information from family folders in particular kebele. Individuals who fulfilled the inclusion criteria of FGDs and asked to give consent for the interview. Finally, we conducted the interviews with individuals who agreed to participate in the study.

For KIIs, we used criterion sampling. Participants for KIIs were recruited from governmental and non-governmental organizations as well as from community. We recruited them in collaboration with heads of weredas Health Office, HEWs and kebele leaders in the selected weredas.

### Operational definition of husband involvement

There is no universal definition of husbands’ involvement in maternal health care [26]. According to USAID (2009) definition, husbands’ involvement in maternal health care as men presences physically with his pregnant wife in health facility to give her physical, emotional and financial support that enables her to access the routine ANC, delivery care and PNC services from skilled providers [2].

For this study ‘husband’s involvement’-is defined as when a husband accompany his pregnant wife to the health facility for at least one ANC visit, or delivery care or PNC visit but not for his medical problem’.

### Data collection tools and procedures

Data were collected using interviewer’s guide questions, which were developed, first in English language, then translated to Amharic language, and then translated back to English language. This was done to reduce any confusion on meanings of terms in the questions. A 3 days training about the overall objective and procedures of the study, moderating skills on data collection techniques with role-plays, and a one-day field practice in the nearest one kebele were done for data collectors and supervisors by P.I.

Informed written consent for the study was obtained from all eligible participants who agreed to participate before we commenced data collection. The interview guide included four parts. Part I and II were FGD guide questions for men and women, respectively. Part III was IDI guide questions, and part IV was KII guide questions. The interview guides included seven to ten broad questions with suggested probes. Trained facilitators (two males and two females) who were supported by note-takers conducted the FGDs. The facilitators have had a master degree in health related fields and a previous experience of facilitating FGD or IDIs. While the female moderators conducted the women’s FGDs and IDIs, the male moderators conducted the men’s FGDs and IDIs. The locations of FGDs were prepared in consultation with the kebele leaders and health extension workers (HEWs). For IDIs, the locations were determined by the research team with consideration given to potential sensitivities relevant to each participant. KIIs were conducted in the informants’ offices of selected organizations. The discussions and interviews were conducted in quiet rooms, and privacy was ensured to enable participants and interviewees to feel free and express their opinions. The interviews and FGDs were scheduled two or 3 days ahead of time in consultations with the participants. During fieldwork, besides taking field notes on face-to-face discussions, a tap-recorder was used for participants who gave consent for recording. On average, an hour time was given for each KII and IDI. Whereas each FGD comprised between eight and twelve participants of the same sex and took approximately 1 h and 30 minutes to 2 h [27].

### Data analysis

Tape-recorded data were transcribed and extensive field notes of interviewers were reviewed on daily base. Transcriptions were made word by word (verbatim) by interviewers and principal investigator. All Focus Group discussions and IDIs were conducted in Amharic and Sidama-Afoo languages based on the participants’ choice. The transcribed data were translated into English. The data were uploaded into an ATLAS.ti7 software program for coding purpose. The coding was done by two individuals separately. Using an ATLAS.ti 7 training manual, analysis were done using the four phases of theme development: initialization, construction, rectification and finalization**.** First, open codes were created by reading the transcripts line by line; and then, words with similar meanings were grouped into categories. In the next step, selective coding was performed and relevant codes were further categorized to form themes. Prominent themes were then further categorized to sub-themes. In addition, direct quotes and narrations from participants were reported without editing to avoid losing contextual meaning of important issues [28].

## Results

### Participants

A total of 12 IDIs, 10 KIIs, and 10 FGDs (5 FGDs with men and 5 FGDs with women groups) were conducted in the selected five Weredas of Sidama zone. (Table 1).

**Table 1:** Background characteristics of participants for focus group discussions, in-depth interviews, and key informants’ interviews, in five woredas of Sidama zone, southern Ethiopia, 2015

Characteristics		10 FGD		12 IDI		10KIIs	
		Female (*n* = 42)	Male (*n* = 38)	Female (*n* = 5)	Male (*n* = 7)	Female (*n* = 4)	Male (*n* = 6)
Age (year)	18–30	19	16	2	3	1	0
	> 30	23	22	3	4	3	6
Education status	<Primary (1–6 grade)	35	22	4	2	0	2
	>Secondary (7–12 grade)	7	16	1	5	4	4
Occupation type	Government employee	8	24	2	5	3	3
	NGO	0	4	0	1	1	1
	Private	3	9	0	1	0	2
	Housewife	31	–	3	–	0	–
	Unemployed	–	1	–	0	–	0

*FGD* Focus group Discussion; *IDI* In-depth Interview; *KII* Key-Informant Interview; *NGO* Non-governmental organization

Majority of the participants in this study agreed on the low level of husbands’ involvement in maternal health care in their areas. However, they mentioned that majority of the husbands accompanied their wives to health facilities for labour and birth care compared to ANC and PNC visits. The participants also reported multiple factors for low husbands’ involvement in maternal health care. These factors are categorized in to four sub-themes: childbirth is a natural process, pregnancy and childbirth are women’s business, preference of TBAs’ care and novelty of the concept of husband’s involvement in pregnancy and birth care.

### Childbirth is a natural process

Participants in FGDs and IDIs mentioned that peoples’ view of childbirth had its own part to prevent the involvement of husbands in maternal health care. They explained that many men believed birth as a natural process that can be managed without medication at home with the help of TBA.

A 43 year-old man explained: *“Many husbands preferred home deliver for their wives, I mean…uh…..uh…. unless a woman gets sick during pregnancy, they do not want to go to Hospital. Since, they considered childbirth as a natural process that should be endured at home according to local practices.”*

A 47 years man expressed this, as follow: *“I have eight children, and all the births were conducted at home with the help of a birth attendant [TBA]. Since, it [home birth] is descended from our parents; you know….they brought [birth] us to this world by the same process. They didn’t know about any medical treatment for pregnancy and birth; they believed that it is a natural process for human and all mammal! They [our parents] always said that it is given by God; no one stop this process!”*

Participants in the KIIs also mentioned that the perceived social norms by the society guide their choice for place of childbirth. The preference of home birth than birth in health facility, prevent, not alone men to involve in maternal health care, it also prevent women to get skilled care at health facility.

Many husbands in this study expressed that men in the old generation described the accustomed experiences from their parents and the pressure from other family members, including their wives, for a home birth.

A 45-years man participant reported, *“If my wife is not sick, we prefer home birth; I mean, home is better for her. Because, our father and mother did the same thing!”*

### Pregnancy and childbirth are women’s business

The perceived social norm was also expressed in terms of ‘gender roles’ in pregnancy care and childbirth. It was reported as a reason for lack of husbands’ involvement in their wives’ maternal health care. Many participants believed that women and men have different roles in the provision of care for pregnant women and laboring women. They mentioned that providing body care for pregnant woman and performing house chores are the roles of women, whereas men’s role during pregnancy and birth are providing physical, emotional and financial supports to his wife.

A 38 years woman reported, *“in our community, it is shame for a man (a husband) to present in women’s place; eh…eh…[?]… and no woman allow to be examined or to give birth in front of men, even her husband, except the professionals. So, let’m ask you one question, what is he doing in women’s clinic? (Others laugh).”*

Many participants from men FGDs mentioned a significant role of husband when his wife is starting labour at home.

A 38 year man expressed as follow, *“A husband is thought to accompany his wife to the hospital. Especially in areas where there is no transport access, he may carry her to hospital on a horseback or an animal-vehicle (Aheya- Gari in Amharic). In addition to that, he is the one who inform the relatives and to call a birth attendant”.*

### Preference for traditional birth attendants’ care

The discussion was generally focused on families’, especially husbands’ feeling on the interactions between laboring mothers and traditional birth attendants versus health care professionals. A common feeling was worry of unkind treatment from health care staffs that put off husbands from the health facilities. The explanations from female participants to the reason why mothers and their husbands might avoid facility-based maternity care were agreed with the explanations from many of the males’ focus group members.

A male participant reported, *“There are people who usually do not attend the clinics; but when they visit the clinic, they are asked to bring their pervious card number. And, if they have not it, this makes them scared! At that time, the nurses are not usually very good compared to the traditional birth attendants. They shout to you, they say bad things to you! So, why we go there! Because, they [doctors and nurses] do not treat people so nicely!”*

On the other hand, majority of the participants expressed their feeling that they are more satisfied by the treatment they got from traditional birth attendants compared with health care providers.

A 32 years woman participant reported, *“The traditional birth attendants encouraged us [mothers] during labor, they cleaned us with warm water after delivery, and they fed and cleaned the newborns! I mean, they just give care as a woman!”*

Many of the participants reported that health workers’ negative attitude towards men involvement during pregnancy and delivery care was another key barrier, which marginalized men and promote the view that pregnancy care is a woman domain.

A 33 years-old female participants expressed: “*Inhospitable and sometimes unreceptive words directed at women and their husbands from few health professionals were a barrier to husbands’ involvement.”*

Similarly, in the males’ focus groups, some dialogue emerged on the reassuring and familiar manner of traditional birth attendants with mothers in the community.

A 28-year-old male participant reported, *“They [Traditional Birth Attendants] are tied to you more than the nurses where there is a challenge.”*

### Novelty of the concept of Husband’s involvement in pregnancy and birth care

Participants reported that many men involved (accompanied their wives to health facility) during labour and childbirth care than during ANC and PNC. Since, childbirth is a more stressful event in pregnancy process. However, almost all the participants agreed that people in their communities including themselves were unfamiliar with the idea of men’s involvement during ANC and PNC. They mentioned that lack of awareness was among the reasons for low men’s involvement in maternal health care.

A 37-year-old male participant reported, *“To tell you the truth, this idea is new for us! We [men] know that pregnancy care is for pregnant women; so, what service a man could obtain there unless wasting his time.”*

Similarly, the participants mentioned that their lack of knowledge and being unfamiliar with the existed services do not allow them to get involved in their wives’ maternal health care.

A 32 years female participant in the FGD expressed this, as follow: *“We are not familiar to such type of service [a maternal health service] that is given to both women and man. It is a new idea for us...[?????]..., and it is copied form outside [foreign] countries.”*

Participants from both men and women’s groups expressed that they do not know the role of a husband in ANC or PNC units. Yet they mentioned that a husband might be called to come with his wife for HIV counseling and testing.

A 31-year-old female participant reported on this, *“I do not know about any examination for man! I mean, there is nothing that men can do in ANC visit, even in labor and birth care, apart from just observing what is happening there.”*

Participants in the FGD repeatedly mentioned that they do not know the reasons why men should come for ANC and PNC services with their wives.

A 43 year-old male participant reported, *“PNC services are for women, why need to come men!, unless to carry the baby; that is also preferred to be done by his/her sisters or our mommy.”*

Participants among the health provider mentioned that only few husbands involved in their wives’ ANC and PNC; and the majority, usually, come for labor and childbirth.

A 36 year-old female midwife reported, *“I guess many factors that contributed for their (husbands’) absence during pregnancy care; for example, lack of information or knowledge about their roles during ANC and PNC visits, and due to lack of communication between spouses.”*

A 36 years-old men (health provider) mentioned, *“we were not working on this agenda due to different reasons. Among them, absence of guideline and lack of trained professionals were made the health professionals unable to work with men during ANC, delivery and PNC visits. They [including us] do not know about what roles a husband can play by his presence during ANC, delivery or PNC.*

A 29 years female midwife added on this, *“There should be a written guideline for male involvement in maternal health care in each health facility; and well trained professionals should give proper orientation to both health providers and the community.”*

Similarly, a health officer reported that there is no guideline or standards of practices [SOPs] about male involvement in maternity care services at health centers as well as at hospital levels. He also explained his doubt on its existence even at national [Ministry of health] level.

A 38 years-old male health officer exclaimed, *“Let me ask you a question, if a husband enters to ANC or delivery room, what is his role unless to make a room more crowded? Actually, I have known few private hospitals, which have started a couple’s counseling sessions during ANC examinations or before CS [Caesarean Section], especially for ultrasound examination and others intensive diagnostic tests. That is also another thing! I mean, it is related to some sort of benefits to the hospital.*

## Discussion

This study presented in-depth insight into a range of factors related to low husbands’ involvement in antenatal and postnatal care.

The concept of men accompanying wives to ANC and PNC services is not yet accepted in the study communities. This is due to socially constructed norms, particularly, gender role norms and traditional view of childbirth, and the foreign nature of the concept of male engagements in maternal care. Social norms are “... expectations held by social groups that dictate appropriate behavior and are thought of as rules or standards that guide behavior” [29]. In this study, we recognized the influence of existing socially constructed norms particularly gender norms and traditional view of childbirth on the type of support a husband provides to his wife during pregnancy, birth, and after birth. This finding is supported by other studies that mentioned husbands’ perceived social-norm is an important factor that influence his wife health care utilization as well as his involvement in the services because of his close social relationships and decision-making power within the household [6, 7]. With regard to gender roles, particularly in the patriarchal system, men’s role is predominantly in the public sphere of production and politics, while women’s is in the domestic, household and child rearing [5]. This implies that the social norm that a woman in labor should be cared by women is entirely entrenched in the study areas [6, 7, 30–36].

Individuals or communities in the traditional views were generally categorized as *old generation group*. This could be due to lack of information, education and communication in392 the country, especially in the last 10 to 20 decade.

In this study, women themselves were one of the barriers to husbands’ involvement in maternal health care. Women participants in this study expressed that some women were reluctant to be with their husbands at health facility for ANC and PNC. Similar results have been documented in earlier studies done in Africa countries [6, 34]. The study reported that often women are embarrassed to be with their husbands in maternal health care services; since, ANC services and other maternity care services are seen as “female” places, which designed and reserved for women. Interestingly, some of the women reported that they do not like to be seen with their male partner attending ANC [34]. In addition, women mentioned that the presence of husbands at health care units makes difficult for some women to express their feeling or opinion freely and honestly. Thus, women who do not want their husband to accompanying them to ANC or PNC would not communicate to them when the health care providers requested women to come with their husbands during the subsequent antenatal care visit [6]. This implies that husbands’ participation was depended upon the relationship that exists among the couples.

Lack of awareness about husbands’ involvement during maternal health care was one important barrier for their involvement in their wives’ ANC, delivery care and PNC. In this study, almost no respondents had known that men are expected to involve in their wives’ ANC and PNC services. Similar findings in Africa and Asia have shown that some men expressed ignorance and others did not understand why they had to be involved [6, 9, 31, 37–39]. A study in Cambodia reported that many men are not aware of why they need to be involved in SRH, how they can be involved and what services are available for them and their partner [38]. Moreover, studies in Malawi and Kenya indicated that men’s retained the negative health beliefs from the overall lack of knowledge about maternal health care may attributed to husbands’ lack of involvement in maternal health care [6, 40]. One possible reason for this could be due to the absence of men-or-couples oriented community-level reproductive health campaigns. Similarly, the finding of current research indicates a need to educate men in the public sphere with appropriately tailored health messaging to attract towards RH services [40]. This implies that educating men about male’s involvement in maternal health care is a crucial for their participation in the care.

With regard to health workers’ negative attitude, the way in which maternity care was provided to mothers was an important factor that prevents husbands’ involvement in their wives’ maternity care. ‘The idea that maternal health care is for women’ has been deep-rooted in the majority of health care providers at all levels. Due to this reason, they do not have an initiative to involve husbands in their wives’ health care. They have-not allowed husbands to participate during ANC, delivery care, and PNC counseling, which enhance men’s banishment from the services. Moreover, as we mentioned above, inhospitable and unreceptive words directed at women and their husbands from health professionals discourage men from returning or accompanying their wives to health facilities. This is agreed with the findings of studies in other African countries [6, 34, 39, 40]. In a systematic review of factors affecting use of antenatal care in developing countries, rude and unfriendly attitudes of health care providers were identified as major barriers deterring mothers from delivering in hospital [41–43]. A study in Tanzania expressed the fears of discrimination from health facility staffs as a reason for avoiding hospital deliveries [37]. This implies that the attitudes of health care providers are related to husbands’ participation in maternal health care [37, 41–43]. .

### Study limitations

The findings of this study lacked representativeness; since the country has diverse culture and different utilization rates of skilled health professionals. However, the study sites were selected purposively.

## Conclusions

In general, four main themes, which are childbirth is a natural process, pregnancy and childbirth are women’s business, preference for TBAs’ care and novelty of the idea of husband’s involvement in pregnancy and birth care were identified as barriers for husbands’ involvement in maternal health care, in this study. Therefore, to enable husbands to play more supportive roles in the area of maternal healthcare: *first*, we recommended awareness creation activities about childbirth, on benefits of birth in health facilities and benefits of men’s involvement in pregnancy care; *second*, measures should be taken on health professionals who show bad and un-respectful behaviors towards their clients (both women and men); *third*, encourage TBAs’ services through skilled trainings on pregnancy and birth care; finally, trainings on men’s involvement in maternal health care need to be given for health care professionals at all levels, and awareness creation and information dissemination activities to the people through mass-media as well as mass campaigns. Further, we recommend further research on the effects of quality service on men’ involvement, which was not considered in this study.

## Data Availability

The datasets generated and analyzed during the current study are not publicly available; but are available from the corresponding authors on reasonable request and with permission of the Southern Region and Sidama Zone Health Bureau.

## Abbreviations

ANC: Antenatal Care
CS: Caesarean Section
EDHS: Ethiopia Demographic and Health Survey
FGD: Focused Group Discussion
HEW: Health Extension Workers
HIV: Human Immunodeficiency Virus
IDI: In-depth interview
IMR: Infant Mortality Rate
KII: Key informants’ interview
MMR: Maternal Mortality Ratio
MOH: Ministry of Health
NMR: Neonatal Mortality Rate
PI: Principal Investigator
PNC: Postnatal Care
SNNPR: Southern Nations Nationalities Peoples Region
SOP: Standards Of Practices
TBA: Traditional Birth Attendant
